# How Resource Scarcity and Accessibility Affect Patients’ Usage of Mobile Health in China: Resource Competition Perspective

**DOI:** 10.2196/13491

**Published:** 2019-08-09

**Authors:** Qing Ye, Zhaohua Deng, Yanyan Chen, Jiazhi Liao, Gang Li, Yaobin Lu

**Affiliations:** 1 Department of Information Management Tongji Hospital, Tongji Medical College Huazhong University of Science and Technology Wuhan China; 2 School of Medicine and Health Management Tongji Medical College Huazhong University of Science and Technology Wuhan China; 3 Tongji Hospital, Tongji Medical College Huazhong University of Science and Technology Wuhan China; 4 School of Management Huazhong University of Science and Technology Wuhan China

**Keywords:** mobile health, technology adoption, moderating effect, resource scarcity, resource accessibility, resource competition

## Abstract

**Background:**

The last decade has witnessed many achievements in China’s health care industry, but the industry still faces major challenges among which the uneven distribution of medical resources and the imbalance between supply and demand are the most pressing problems. Although mobile health (mHealth) services play a significant role in mitigating problems associated with health care delivery, their adoption rates have been low.

**Objective:**

The objective of this study was to explore the impact of resource scarcity and resource accessibility on the adoption of mHealth from the perspective of resource competition, to examine the concerning factors, and to provide a theoretical basis for promoting mHealth in China.

**Methods:**

We used 229,516 original registration records of outpatients to conduct an empirical analysis to examine the adoption of mHealth services from the perspective of resource competition.

**Results:**

The adoption rate of mobile services for outpatients was low, accounting for only 31.5% (N=71,707). The empirical results indicated that resource scarcity (beta=.435, *P*=.01) and accessibility (beta=−.134, *P*=.02) have a significant impact on the adoption of mHealth. In addition, gender (beta=.073, *P*=.01) and age (beta=−.009, *P*<.001) are significantly related to adoption of mHealth. Experience with mHealth has a moderating role in the relationship between resource scarcity (beta=−.129, *P*=.02), accessibility (beta=.138, *P*=.04), and adoption of mHealth.

**Conclusions:**

In this study we demonstrate that the external environment (resource scarcity and resource accessibility) has a significant impact on the adoption of mHealth. This study also demonstrates that experience with mHealth has a moderating role in the relationship between the elements of the external environment. Finally, we confirm that mHealth is a key factor in the delivery and allocation of medical resources and provide a theoretical basis for government agencies to develop policies on mHealth.

## Introduction

### Background

In recent years, China’s health care industry has made a series of significant improvements, but it still faces many challenges, including uneven distribution of medical resources, imbalance between supply and demand, etc [1,2]. One particularly enormous challenge lies in ensuring the availability and equal distribution of high-quality resources. Such resources are mainly concentrated in large hospitals located in large or medium-sized cities, which are difficult for patients in rural and remote areas to access [2,3]. The number of physicians and nurses per 1000 persons in China is 2.06 and 2.13, respectively, which is much lower than in developed countries [1].

In addition, in recent years, the use and penetration of mobile phones have grown in China because of the rapid development of technology and the obvious reduction in cost [1,4]. China now ranks among the countries with the highest number of smartphones per capita [5]. With this proliferation of mobile technology, mobile health (mHealth) has become the main driving force in the health care industry. mHealth can be defined as the delivery of health care services through mobile technologies, which mainly comprise online consultations, appointment registration, and health recommendations [6]. Among these apps, appointment registration services, a fundamental and vital channel through which patients seek medical resources, have gained particularly widespread acceptance in China. With the rapid development of mobile technologies and the continuing penetration of mobile devices, mHealth has become a valuable tool and popular option to improve health care and service delivery in underserved regions [7-9]. Chinese government agencies have made policy decisions to support and promote the growth of this important emerging industry [10]. Health care companies and hospitals have begun to pay attention to this issue and have been attempting to provide health services directly to patients through mobile and wireless technologies. These services have the potential to be highly beneficial, especially for patients in rural and remote areas.

Although mHealth presents many opportunities for improving health care delivery, its adoption and use have been low [2,7,11,12]. A robust body of work in information systems has studied factors associated with mHealth adoption and use [11,13-15]. Although extant studies have explored and discussed reasons for these low adoption rates [14,16], empirical research examining this question from the perspective of resource competition is still rare. Previous studies have revealed that a growing number of patients who ordinarily seek health care services offline will move to online channels when medical resources are limited, which will inevitably affect patients’ adoption and use of mHealth [11,15]. However, extant studies of mHealth adoption have mainly focused on patient-related factors, ignoring the impact of the external environment on technology adoption [17,18]. In the context of health care in China, the impact of external factors such as resource scarcity and accessibility caused by lack of and uneven distribution of medical resources on patients’ technology adoption behavior is still unclear.

This paper aims to explore the impact of resource scarcity and resource accessibility on the adoption of mHealth from the perspective of resource competition, to examine the factors involved, and to provide a theoretical basis for promoting mHealth in China. In this research, we pose the following questions:

1. How do resource scarcity and accessibility affect the adoption of mHealth by patients in China?
2. How does patient experience with mHealth moderate the relationship between resource scarcity, resource accessibility, and adoption of mHealth?
3. Does mHealth play a role in the delivery of medical resources?

To answer these questions, we use a dataset of 227,539 outpatient registration records from both online and offline channels to conduct an empirical study. In the next section, we review the related literature. After we construct a conceptual model and develop 4 hypotheses, we present our research method and report our results. Finally, we discuss our research implications and note some limitations.

### Literature Review and Theoretical Background

#### Mobile Health Services

With the rapid development of mobile internet technology and growing prevalence of smartphones, mHealth has become an increasingly practical, innovative approach to health care delivery, especially in rural and remote areas [19-21]. Many applications for mHealth services have been reported in the extant literature, including chronic disease management [22,23], mental health services [24], health solutions for pregnant women and teenage youth [25-27], and health monitoring [28,29].

mHealth overcomes geographical boundaries, enhances the equity and accessibility of medical resources, and provides an effective channel for patients in rural and remote areas to access medical services [30,31]. At present, hospitals provide a variety of services through mobile platforms available in China, which mainly include the WeChat platform and independently developed applications. Services provided comprise online consultation [32,33], appointment registration [7,30,34], electronic medical prescription, and online payment. Among these services, appointment registration is the service most heavily utilized, with which our study is most concerned. Previous studies have shown that experience has a positive significant impact on patients’ adoption of mHealth [13,16,35]. In the context of mobile registration, historical registration records provide us with patients’ experience-related data, affording us an empirical context to demonstrate the moderating effect between resource competition and patients’ adoption of mHealth.

#### Medical Resource Scarcity and Accessibility

Health care issues, which include aging populations, chronic illnesses, rising costs, and access disparities, represent a major challenge in China [3,4,12]. As previously mentioned, the numbers of physicians and nurses per 1000 residents (2.06 and 2.13, respectively in 2014) are relatively inadequate compared with those of developed countries [1]. The shortage and uneven distribution of medical resources is in fact the underlying reason for a number of current health care problems [3].

The finiteness of medical resources results in their scarcity, leading to a supply and demand problem in which the supply of medical resources cannot meet the growing demand of the people, who want access to these knowledge-intensive services when they are sick [36,37]. This results in competition among patients. Obviously, hospitals can only provide limited services to patients every day; thus, only patients who have registered can access medical services. With the development of internet technology and smart devices, patients can now access medical resources via both online (eg, online registration for medical appointments) and offline channels (eg, registered via full service or self-service in a hospital) [30]. Competition for medical resources will lead to behavioral changes. In this context, the online channel is an important supplement to the offline channel, and patients will compete for medical resources via these 2 channels. This change in behavior will affect patients’ adoption of technology. On the basis of these arguments, the impact of medical resource competition on patients’ adoption of technology is very worthy of examination.

#### Extending Channel Complementarity Theory to mHealth Adoption

Technology adoption in health domains has attracted wide attention of scholars and has been studied from many theoretical perspectives such as technology acceptance model, social exchange theory, and identity theories [38-40]. On the basis of channel complementarity theory, this study focuses on the impact of external environment (resource scarcity and accessibility) on technology adoption. Dutta-Bergman constructed channel complementarity theory and suggested the idea of media complementarity [41,42]. Channel complementarity theory argues that individuals will use any available channel to meet their needs [43]. We conceptualize the process of appointment channels selection as an individual information acquisition process by drawing on the theory of channel complementarity. With the development of internet technologies and mHealth, a variety of services channels are emerging. Patients will choose any available channels to obtain needed medical resources, including mobile service channel (online) and traditional channels (offline). The complementary or displacement effects of the channels are manifested under the influence of the external environment. In our research context, the scarcity of medical resources will lead to the competition of patients for medical resources, thus affecting the choice of patients’ service channels and ultimately affecting patients’ technology adoption. In addition, the accessibility of medical resources for patients is varying, which will inevitably affect the choice of service channels and the adoption of mHealth.

### Research Model and Hypotheses

**Figure 1:**
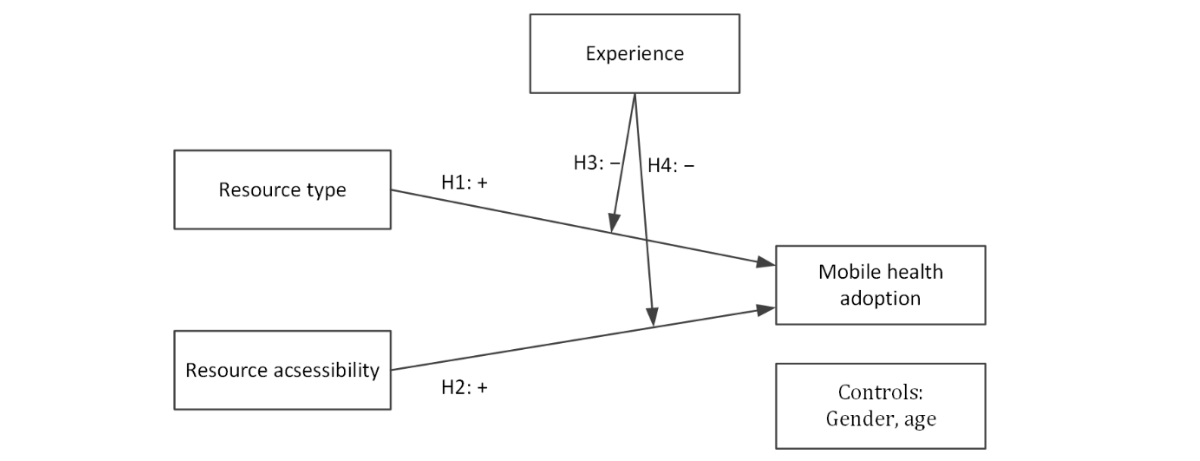
Conceptual model. H: hypothesis.

Extant studies mainly focus on the impact of individual characteristics or technological factors on adoption of mHealth [44-46]. To our knowledge, few studies have considered the impact of the external environment, which may also exert a great influence on patients and their behavior [11,15]. Therefore, we believe that it is necessary to address this gap by studying the impact of the external environment on the adoption of mHealth. In this study, the external environment is characterized in reference to the scarcity and accessibility of medical resources. The scarcity of medical resources is measured according to the patient’s choice of 1 of 2 different physician types, chief physician (higher value, more scarce) or associate chief physician (lower value, less scarce). Accessibility is measured by the distance from the patient’s location to the hospital. In addition, we examine the moderating role of experience with mHealth in the relationship between medical resource scarcity and accessibility and mHealth adoption. The conceptual model of our research is illustrated in Figure 1. The research hypotheses in detail are presented as follows.

#### Medical Resource Scarcity

We define the scarcity of medical resources as limited medical resources relative to people’s diverse health needs. In this study, we use the value and abundance of medical resources to measure resource scarcity. When patients compete for medical resources, they are mainly competing for medical resources of different values. Medical resources of high value are relatively scarce, presenting the problem of unbalanced supply and demand. Patients’ competition for high-value medical resources is more intense, compared with the competition for lower-value medical resources. Mobile registration service provides patients with a very important online channel through which all patients with a smart device can access medical resources. As overall competition intensifies, traditional offline competition will certainly shift to online channels so that patients will compete for medical resources via mHealth platforms. Therefore, we can use medical resource scarcity as a measurement dimension of resource competition to examine the impact of medical resource competition on the adoption of mHealth.

Mobile registration services in China mainly provide 2 types of medical resources, patients may choose to make an appointment with a chief physician or with an associate chief physician. The difference between these 2 practitioner types lies in their level of expertise, so chief physicians are of higher value for patients and competition for appointments with them is more intense. According to channel complementarity theory, patients will meet their medical needs through all channels that can obtain the required medical resources, and mHealth just provides such opportunities and channels. Patients will compete for these 2 types of medical resources through both offline and online channels, including mobile registration. Therefore, the scarcity of resources will affect the channel choice of patients. On the basis of these arguments, we hypothesize the following:

*H1: Medical resource scarcity has a significant positive impact on the adoption of mHealth. The adoption rate of mHealth is high when medical resources have a higher value.*

#### Medical Resource Accessibility

Accessibility of medical resources refers to the degree of difficulty for patients to access medical resources, and distance is an important indicator to measure the accessibility of medical resources. Medical resources are unevenly distributed because of the regional imbalances of economic development in China, which result in lower resource accessibility for many patients. High-quality medical resources are mainly concentrated in large hospitals in big cities, making it difficult for patients in rural and remote areas to access them. As the distance between the patient and the hospital increases, the accessibility of medical resources, especially those of higher quality, will decrease concurrently. In addition to the cost of medical treatment, more remote patients face additional costs such as transportation and accommodations. We hypothesize that to reduce these associated costs, patients who are far away from the hospital will generally register via mHealth service and confirm that the registration is successful before they go to the hospital.

According to the channel complementarity theory, patients with lower accessibility of medical resources will show channel displacement effect, that is, they will reduce medical costs through the mobile channel. Therefore, we can safely assume that the accessibility of medical resources may affect patients’ adoption of mHealth. Distance to the hospital can be used as a measure of medical resource accessibility and can be used to examine the impact of accessibility of medical resources on the adoption of mHealth. In this context, we believe that the accessibility of medical resources is low for patients who are far away from the hospital. To compensate for this disadvantage, patients must use mHealth services as a tool to obtain medical resources. Thus, we formulate the following hypothesis:

*H2: The accessibility of medical resources associated with mHealth is negatively related to patient adoption of mHealth. Patients who must travel longer distances to a hospital have a higher mHealth adoption rate.*

#### The Moderating Effect of Experience

Existing studies have shown that experience with mHealth has a positive impact on its adoption [7,13,47,48]. In this study, we agree with this argument and will further confirm it in the following empirical section. In doing so, we draw on the attribution theory, which argues that people tend to attribute their actions to the conditions of the external environment and as a result, they may limit their actions [49,50]. In the context of our research, the scarcity and accessibility of medical resources are the external environmental factors that may limit patients’ adoption of mobile services, and the experience of success or failure with mobile registration will affect patients’ attribution. Attribution of previous successes or failures in a specific action (in our case, mobile registration) will affect patients’ expectations, emotions, and efforts the next time they have the opportunity to use mobile registration. Patients’ experiences with mobile registration are often unsuccessful or may even fail because of the scarcity of and competition for medical resources. This unsuccessful or failed experience has a certain impact on the relationship between resource scarcity, accessibility, and adoption of mHealth. Therefore, it is necessary to study the moderating role of patient experience in the relationship between resource scarcity, accessibility, and the adoption of mHealth. In our research context, we argue that this moderating effect is negative. On the basis of the arguments above, we propose the following hypotheses:

*H3: Experience with mHealth has a negative moderating role in the relationship between medical resource scarcity and patients’ adoption of mHealth.*

*H4: Experience with mHealth has a negative moderating role in the relationship between accessibility of medical resources and patients’ adoption of mHealth.*

## Methods

### Research Context

**Figure 2:**
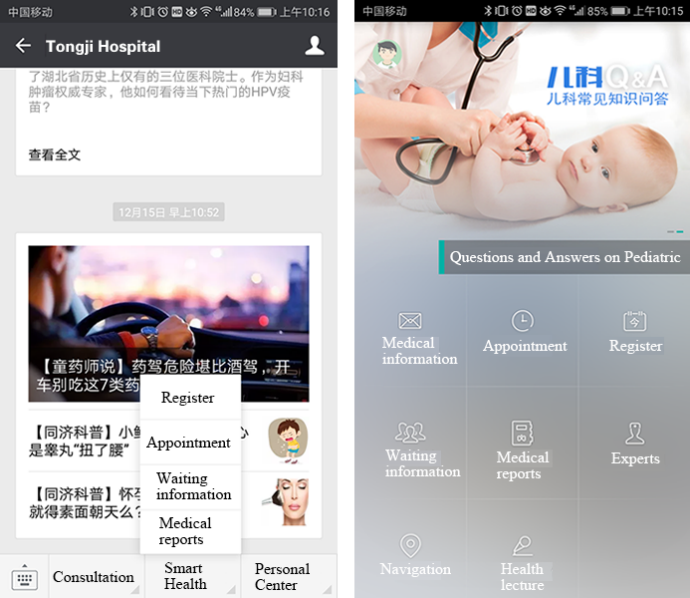
Tongji hospital registration snapshots from the WeChat platform and E-Tongji app.

In this study, our research environment is Tongji Hospital, which is a large, tertiary, multispecialty referral hospital in China. Founded in 1900 by Dr Erich Paulun, a German physician, Tongji Hospital is an innovative modern hospital integrating medical care, teaching, and research [51]. In addition, Wuhan Tongji Hospital is one of China’s top 10 most well-known hospitals, according to Fudan rankings. Tongji Hospital has offered a mobile online appointment registration service since 2014, utilizing platforms such as WeChat, the E-Tongji app, and some third-party registration platforms such as Guahao website and the China Mobile 12580 platform (see Figure 2 for snapshots of the WeChat platform and E-Tongji app). Tongji Hospital offers traditional offline registration channels as well. The hospital provides 2 types of medical resources, chief physician and associate chief physician, which, as mentioned above, are accorded different values, as patients in China tend to value chief physicians over associate chief physicians. Both resource types are in short supply, providing a suitable environment for us to study the impact of resource scarcity on the adoption of mHealth. Patients in Tongji Hospital come from many provinces in China, providing us with a wide range of distance to hospital measurements, which we use to gauge the accessibility of medical resources for each patient.

### Sample and Data Collection

In our research, we used 4 specific datasets. The first comprised 229,516 original registration records of outpatients from both online and offline channels. The registration records were collected from the appointment and registration platform of the Optical Valley branch of Wuhan Tongji Hospital between June 2016 and May 2017. All registration records were anonymized in accordance with patient privacy regulations. Each registration record contains patient’s age, patient’s gender, type of medical resource (chief physician or associate chief physician) selected, a variable for prior experience with mobile registration, and the first 7 digits of the patient’s mobile phone number. The second dataset contains the travel distance between the city in which the patient is located and the Optical Valley branch of Wuhan Tongji Hospital, as calculated by Baidu Map. The third dataset was used to obtain patients’ location by linking patients’ mobile phone numbers to publicly available city information. A patient’s city of residence was deduced based on the first 7 digits of their mobile phone number (similar to what one can do using area codes in the United States). Any incomplete or erroneous records were removed to ensure the reliability of the results. Of the 229,516 original registration records, a total of 227,539 (99.14%) records were analyzed after screening.

### Variables and Models

Our research variables are presented in Table 1. Gender and age of patient are control variables. The independent variables are experience, resource scarcity, and resource accessibility. Experience with mHealth is identified according to whether the patient has previously used a mobile registration service. Resource scarcity is measured by medical resource type (chief physician or associate chief physician). In a microenvironment (a specific hospital), there are generally fewer physicians with higher title than those with lower title, which indicates that they are more scarce. Resource accessibility is measured by distance from patient location to the hospital. Adoption of mHealth, which we define as patient registration via any of the several mHealth service channels, serves as the dependent variable. The available mHealth service channels include the WeChat platform, E-Tongji app, and some third-party registration platforms.

**Table 1:** Definition of variables.

Variable		Definition and measurement	Symbols
**Dependent variable**			
	Adoption	A binary variable was used to measure whether the patient made an appointment via mobile health service; 0=never, 1=yes	—^a^
**Independent variables**			
	Resource scarcity (RS)	RS is measured by the type of medical resource; 0=associate chief physician, 1=chief physician	RS
	Resource accessibility (RA)	RA is measured by distance to the hospital; 1=less than 300 km, 0=more than 300 km. 300 km is the distance from the farthest city in the province to the city where the hospital is located	RA
	Experience (EXP)	Whether patients have experience with mobile registration service; 0=no, 1=yes	EXP
	Gender	Value 0=a male patient and value 1=a female	—
	Age	Patient age in years	—

^a^Not applicable.

We conducted logistic regression using SPSS version 23.0 to test our research hypotheses. For this study, our analysis comprises 3 steps. In the first step, we estimate our model using only control variables. Second, we add the main effect, including experience, resource scarcity, and resource accessibility. Finally, we incorporate the interaction effect into our model. The full empirical model can be expressed as follows:

logit (*Y*_i_)

= β_0_+ β_1_ Gender_i_+ β_2_ Age_i_+ β_3_ Experience_i_

+ β_4_ ResourceScarcity_i_+ β_5_ ResourceAccessibility_i_

+ β_6_ ResourceScarcity_i_×Experience_i_

+ β_7_ ResourceAccessibility_i_×Experience_i_+ε_i_

where β_0_ is the constant term. β_1_ and β_2_ are the coefficients associated with control variables. β_3,_ β_4,_ and β_5_ are the coefficients associated with examined variables. β_6_ and β_7_ are the coefficients of interaction terms. Gender_i_, Age_i,_ and Experience_i_ represent patient characteristics. ResourceScarcity_i_ and ResourceAccessibility_i_ account for the external environment of medical resource competition. ε_i_ is the error term.

## Results

### Descriptive Study

The descriptive statistics and correlations for variables in our study are shown in Table 2. The adoption rate of mobile services for outpatients was low, accounting for only 31.5% (N=71,707) and the mean age of outpatients was 37.109 years (SD 18.987). The results indicate that the dependent variables are correlated with the independent variables, and the correlation between study variables and control variables is low. These results show that multiple collinearity is not a significant problem in our research, which guarantees the accuracy of the model estimation.

**Table 2:** Descriptive statistics and correlation matrix (N=227,539).

Variable number	Descriptive statistics construct	Mean (SD)	Variable number				
			1	2	3	4	5
1	Age of patient	37.109 (18.987)	1.000^a^	—^b^	—	—	—
2	Gender of patient	0.549 (0.498)	0.058^c^	1.000	—	—	—
3	Experience	0.193 (0.395)	−0.027^c^	0.031^c^	1.000	—	—
4	Resource scarcity	0.510 (0.499)	0.009^c^	−0.009^c^	0.033^c^	1.000	—
5	Resource accessibility	0.107 (0.309)	0.009^c^	0.020^c^	0.012^c^	0.006^d^	1.000
6	Adoption	0.315 (0.465)	−0.077^c^	0.019^c^	0.093^c^	0.010^c^	0.294^c^

^a^Correlations between 2 variables are calculated using Pearson correlation analysis.

^b^Not applicable.

^c^*P*<.001.

^d^*P*<.005.

### Empirical Results

The regression results are presented in Table 3. Model 1 includes only control variables— *gender* and *age* —which are both significantly related to the adoption of mHealth. Specifically, patient age has a significant negative impact on the adoption of mHealth and patient gender has a significant positive relationship to the adoption of mHealth. Model 2 includes the main effects, in addition to the control variables. We find that all the main effects’ coefficients are significant. Experience with mHealth and medical resource scarcity are significantly positively related to the adoption of mHealth, whereas medical resource accessibility is significantly negatively related. Model 3 includes the interaction terms, in addition to the main effects and control variables. In model 3, all the main effects’ coefficients are significant, which is consistent with model 2. The interaction effects have significant negative coefficients.

**Table 3:** Regression results.

Variable	Model 1	Model 2	Model 3
Constant	−0.504 (0.011)^a^	−1.045 (0.013)	−1.067 (0.013)
Age of patient	−0.009^b^ (0.000)	−0.009^b^ (0.000)	−0.009^b^ (0.000)
Gender of patient	0.102^b^ (0.009)	0.073^b^ (0.010)	0.073^b^ (0.010)
Experience with mobile health (EXP)	—^c^	1.484^d^ (0.011)	1.568^d^ (0.017)
Resource scarcity (RS)	—	0.404^b^ (0.010)	0.435^d^ (0.011)
Resource accessibility (RA)	—	−0.104^d^ (0.015)	−0.134^d^ (0.017)
RS×EXP	—	—	−0.129^d^ (0.022)
RA×EXP	—	—	0.138^d^ (0.037)
Log-likelihood	282,093.080	262,017.222	261,970.485
Cox and Snell R-square	0.007	0.090	0.091
Nagelkerke R-square	0.009	0.127	0.127

^a^The values in parentheses are standard deviation.

^b^*P*<.01.

^c^Not applicable.

^d^*P*<.05.

In H1, we posited that medical resource scarcity has a significant positive impact on the adoption of mHealth, which occurs at a high rate when medical resources have a higher value. As shown in Table 3, the coefficients of medical resource scarcity are positive and significant in model 2 (beta_4_=.404, *P*<.05) and model 3 (beta_4_=.435, *P*<.05). Therefore, H1 is supported. In H2, we hypothesized that the accessibility of a medical resource associated with mHealth is negatively related to the adoption of mHealth, so that patients who must travel a greater distance to the hospital would have a higher adoption rate of mHealth. As shown in Table 3, the coefficients of medical resource accessibility are negative and significant in model 2 (beta_5_=−.104, *P*<.05) and model 3 (beta_5_=−.134, *P*<.05). Thus, hypothesis H2 is supported. In H3 and H4, we posited that experience with mHealth has a negative moderating role in the relationship between medical resource scarcity and patients’ adoption of mHealth, whereas medical resource accessibility has a positive moderating effect. The interaction effects’ coefficients are significant in model 3 (beta_6_=−.129, *P*<.05; beta_7_=.138, *P*<.05), thus supporting hypotheses H3 and H4. In the next section, we will test the robustness of our models.

### Robustness Check

**Figure 3:**
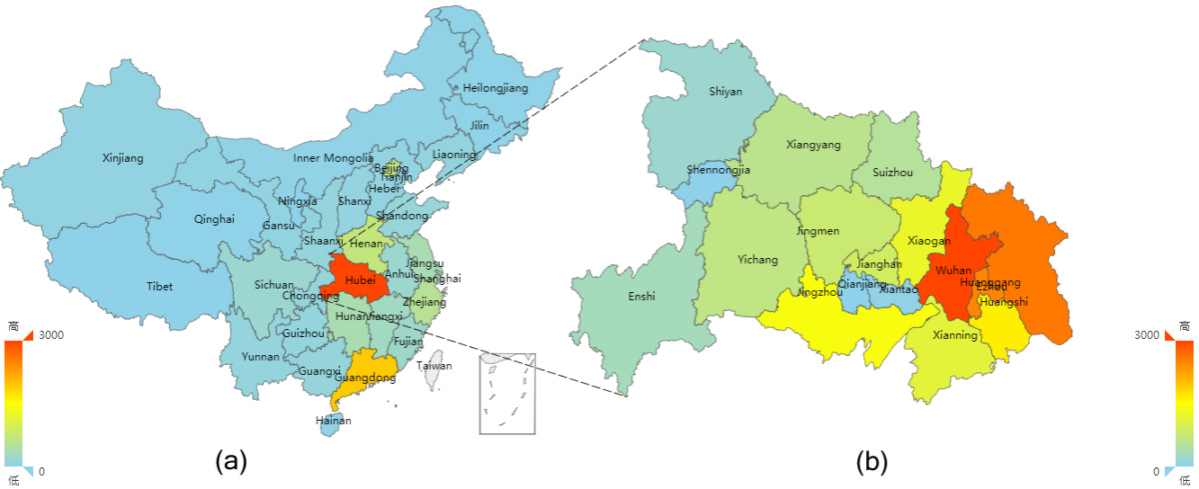
Geographical distribution of patients who register online.

To obtain a more fine-grained understanding of our sample, we conducted visualization analysis of the geographical distribution and registration channels of outpatients. In our sample, 206,327 of 227,539 outpatients (90.68%) who registered through both online and offline channels were from Hubei Province. To demonstrate the geographical distribution characteristics of the patients who used online channels, we identified the number of patients by distinct colors at the national and provincial level. The visualization results are shown in Figure 3. At the national level (see Figure 3) and among those patients who registered online, most patients (N=65,760, 91.71%) came from Hubei Province, followed by Henan and other neighboring provinces. Within Hubei Province (see Figure 3) and among those patients who registered online, a total of 53,047 (80.67%) patients came from Wuhan, with Ezhou (N=2328, 3.5%) ranking second and Huanggang (N=2181, 3.3%) ranking third. This visualization shows that at both the national and provincial levels, the number of patients who used online channels gradually decreased as the distance to the hospital increased.

**Figure 4:**
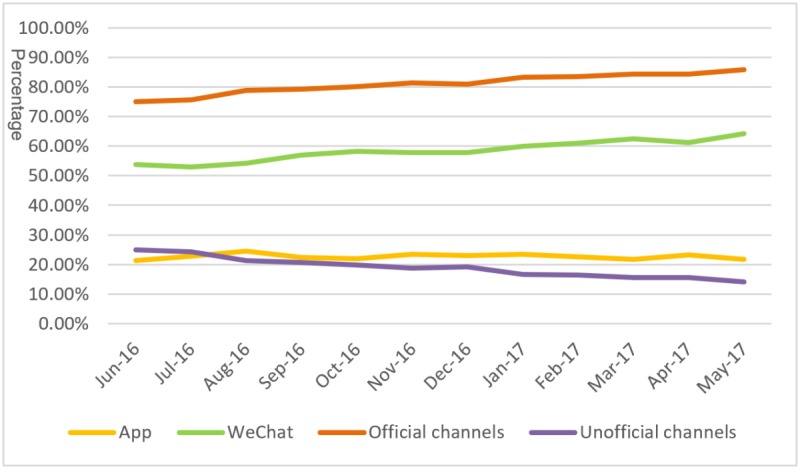
Trend in the proportional use of registration channels.

We also examined the online channels used for registration, with Figure 4 displaying the trends in proportional use of each channel. This graph shows that the WeChat platform displays an upward trend in proportional use each month between June 2016 and May 2017, whereas the use of the E-Tongji app decreases. Furthermore, the proportional use of the WeChat platform is higher than the E-Tongji app in all months. WeChat and the E-Tongji app constitute the official registration channels provided by the hospital directly, but in addition, unofficial channels, such as Guahao website and the China Mobile 12580 platform, constitute an intermediary platform. During our study period, the proportional use of official channels increased from 75.02% to 85.89%, whereas the proportional use of unofficial channels decreased from 24.97% to 14.11%.

On the basis of Figures 3 and 4, we find that most of the hospital’s patients are concentrated in Hubei and that channel selection is relatively stable. The likely reason is that each province has its own medical resources. Therefore, we narrow our sample so as to only consider patients from Hubei and then test the stability of the models. In the robustness check, we incorporate medical resource accessibility as a continuous variable into the models, whereas other variable definitions remain unchanged. The results, presented in Tables 4 and 5, indicate that our models are stable when we control for the impact of geographical factors.

**Table 4:** Descriptive statistics and correlation matrix (N=206,327).

Variable number	Descriptive statistics construct	Mean (SD)	Variable number				
			1	2	3	4	5
1	Age of patient	37.170 (19.148)	1.000^a^	—^b^	—	—	—
2	Gender of patient	0.553 (0.497)	0.055^c^	1.000	—	—	—
3	Experience	0.194 (0.396)	−0.029^c^	0.028^c^	1.000	—	—
4	Resource scarcity	0.511 (0.499)	0.009^c^	−0.011^c^	0.034^c^	1.000	—
5	Resource accessibility	70.149 (60.985)	0.061^c^	0.001^e^	−0.022^c^	0.015^d^	1.000
6	Adoption	0.314 (0.464)	−0.081^c^	0.017^c^	0.297^c^	0.094^c^	0.018^c^

^a^Correlation coefficients between 2 variables are Pearson correlation coefficients.

^b^Not applicable.

^c^*P*<.001.

^d^*P*<.005.

^e^Not significant.

**Table 5:** Regression results.

Variable	Model 1	Model 2	Model 3
Constant	−0.493 (0.011)^a^	−1.095 (0.014)	−1.124 (0.015)
Age of patient (Age)	−0.009^b^ (0.000)	−0.009^b^ (0.000)	−0.009^b^ (0.000)
Gender of patient (Gender)	0.093^b^ (0.010)	0.066^b^ (0.010)	0.066^b^ (0.010)
Experience with mobile health (EXP)	—^c^	1.498^d^ (0.012)	1.621^d^ (0.022)
Medical resource scarcity (MRS)	—	0.403^b^ (0.010)	0.430^d^ (0.012)
Medical resource accessibility (MRA)	—	−0.001^b^ (0.000)	−0.001^b^ (0.000)
MRS×EXP	—	—	−0.112^d^ (0.024)
MRA×EXP	—	—	0.001^b^ (0.000)
Log-likelihood	255,344.365	236,735.682	236,692.326
Cox and Snell R-square	0.007	0.093	0.093
Nagelkerke R-square	0.010	0.130	0.130

^a^The values in parentheses are standard deviation.

^b^*P*<.01.

^c^Not applicable.

^d^*P<*.05.

## Discussion

### Principal Findings

The goal of this research was to explore the impact of external environment (resource scarcity and resource accessibility) on the mHealth adoption and to identify the moderating role of experience. Drawing from channel complementarity theory and attribution theory, we proposed our research hypotheses and conducted empirical research.

The empirical results of our study support all our hypotheses and confirm some previous arguments. In line with previous work, our results indicate that age is negatively and significantly related to the adoption of mHealth services, with young people showing higher acceptance than the elderly [2,13]. Extant studies have also demonstrated that gender is strongly associated with mHealth adoption [7,52]. In our research, we also identify a statistically significant difference in mHealth adoption between women and men. Overall, women exhibit more positive attitudes toward mHealth services, which has been rarely reported before. One possible explanation is that as the popularity of mobile devices has increased, the technological knowledge gaps between men and women have narrowed, and women are more likely to handle the task of making doctor’s appointments for other family members. Existing studies have shown that experience with mHealth is a crucial factor determining the use of mHealth [48], which is also confirmed by our study. Our results reveal that experience has a significant and positive impact on the adoption of mHealth.

Based on the main effect of our model, empirical results demonstrate that the external environment, characterized here as an environment of medical resource scarcity, has a positive and significant impact on the adoption of mHealth, whereas medical resource accessibility is negative and significant. In our study, medical resource scarcity is assessed based on which medical resource type (chief physician or associate chief physician) a patient selected. These different physician types are of different values to patients, and the fact that patients in China place a higher value on chief physicians and that their numbers are relatively limited both prompt patients to move from offline to online registration to compete for this limited medical resource. Therefore, we observe that resource scarcity is positively and significantly related to the adoption of mHealth. In terms of accessibility, which we measure using the distance from the patient’s location to the subject hospital, we find that resource accessibility has a negative and significant impact on the adoption of mHealth. Patients have a higher mHealth adoption rate when medical resource accessibility is low. This result shows that mHealth services play a significant role in the delivery of medical resources and improve the fairness and accessibility of health care in rural and remote areas. Finally, considering the interaction effect in our model, the results indicate that experience with mHealth has a negative moderating role in the relationship between medical resource scarcity and patients’ adoption of mHealth, whereas medical resource accessibility has a positive moderating effect.

### Contributions

Our results contribute to the existing literature in several ways. First, we addressed the research gap previously discussed, noting that existing studies about technology adoption have focused mainly on patient-related factors rather than external environments [15,16]. On the basis of channel complementarity theory and attribution theory, we conducted an empirical study to analyze the adoption of mHealth from the perspective of resource competition and found that medical resource scarcity and accessibility have significant impacts on the adoption of mHealth. Second, we found that patients have a higher mHealth adoption rate when they must travel greater distances to the hospital. We therefore argue that mHealth has a significant role in health care delivery and medical resource allocation [1]. Finally, we confirmed that patients’ prior experience with mHealth is positively and significantly related to the adoption of mHealth, and we found that experience has a moderating effect in the relationship between resource scarcity, accessibility, and adoption of mHealth.

We also made several useful contributions to the practice and policy surrounding mHealth. As it establishes the important role of mHealth in the delivery and allocation of medical resources, our work provides a theoretical basis for government agencies to develop policies on mHealth. For health care providers and stakeholders, we propose several feasible measures to increase the mHealth adoption rate, which will maintain their advantage and competitiveness in the health care market. Our results also suggest that health care providers and policymakers can take measures to encourage patients to use mHealth services and therefore increase the overall adoption rate, such as cultivating habitual patient use of mHealth services, reducing online registration fees, and taking mobile channel priority.

### Limitations

There are some limitations to our study. First, as the data were provided by one specific hospital, our results may be influenced by this hospital’s particular characteristics, and the universal adaptability of our results may be limited. Second, we only used age and gender as our control variables, so we may be lacking some patient-related variables, such as education, income level, and technological competence, which could affect our results. Third, in this study, we used 2 different physician types to measure medical resource scarcity, both of which are expert resources; however, outpatient medical resources in China also include attending physicians, which would be considered more of a general medical resource. In this study, we did not examine the differences between expert and general resources, which could potentially be significant. We plan to incorporate this distinction and further enrich our results in future research.

### Conclusions

In summary, our study confirms that gender, age, and experience are significantly related to the adoption of mHealth, findings which are in line with existing studies. Our empirical results reveal that medical resource scarcity and accessibility have positive and negative impacts, respectively, on the adoption of mHealth. Experience with mHealth plays a moderating role in the relationship between resource scarcity, accessibility, and adoption of mHealth. We hope that further research will enrich our understanding of mHealth adoption and the impact of external environmental factors.

## Abbreviations

mHealth: mobile health

## References

[1] Zhang X, Lai K, Guo X (2017). Promoting China's mHealth market: a policy perspective. Health Policy Technol.

[2] Sun J, Guo Y, Wang X, Zeng Q (2016). mHealth for aging China: opportunities and challenges. Aging Dis.

[3] Yip W, Hsiao W (2014). Harnessing the privatisation of China's fragmented health-care delivery. Lancet.

[4] Stefanacci RG, Riddle A (2017). China - A half billion older adults getting and giving assistance. Geriatr Nurs.

[5] Newzoo.

[6] Zhang X, Guo X, Wu Y, Lai K, Vogel D (2017). Exploring the inhibitors of online health service use intention: a status quo bias perspective. Inf Manage.

[7] Hoque MR (2016). An empirical study of mHealth adoption in a developing country: the moderating effect of gender concern. BMC Med Inform Decis Mak.

[8] Hamilton EC, Saiyed F, Miller CC, Eguia A, Fonseca AC, Baum GP, Tsao K, Austin MT (2018). The digital divide in adoption and use of mobile health technology among caregivers of pediatric surgery patients. J Pediatr Surg.

[9] WHO Global Observatory for eHealth (2019). Mhealth: New Horizons For Health Through Mobile Technologies (Global Observatory For Ehealth).

[10] Wu H, Deng Z (2019). Knowledge collaboration among physicians in online health communities: a transactive memory perspective. Int J Inf Manage.

[11] Chib A, van Velthoven MH, Car J (2015). mHealth adoption in low-resource environments: a review of the use of mobile healthcare in developing countries. J Health Commun.

[12] Henriquez-Camacho C, Losa J, Miranda JJ, Cheyne NE (2014). Addressing healthy aging populations in developing countries: unlocking the opportunity of eHealth and mHealth. Emerg Themes Epidemiol.

[13] Hoque R, Sorwar G (2017). Understanding factors influencing the adoption of mHealth by the elderly: an extension of the UTAUT model. Int J Med Inform.

[14] Rai A, Chen L, Pye J, Baird A (2013). Understanding determinants of consumer mobile health usage intentions, assimilation, and channel preferences. J Med Internet Res.

[15] Maiga G, Namagembe F (2014). Predicting adoption of mHealth technology in resource constrained environments.

[16] Khatun F, Heywood AE, Ray PK, Hanifi SM, Bhuiya A, Liaw S (2015). Determinants of readiness to adopt mHealth in a rural community of Bangladesh. Int J Med Inform.

[17] Kaphle S, Chaturvedi S, Chaudhuri I, Krishnan R, Lesh N (2015). Adoption and usage of mHealth technology on quality and experience of care provided by frontline workers: observations from rural India. JMIR Mhealth Uhealth.

[18] Huang H, Bashir M (2017). Users' adoption of mental health apps: examining the impact of information cues. JMIR Mhealth Uhealth.

[19] Banos O, Villalonga C, Garcia R, Saez A, Damas M, Holgado-Terriza J, Lee S, Pomares H, Rojas I (2015). Design, implementation and validation of a novel open framework for agile development of mobile health applications. Biomed Eng Online.

[20] Silva B, Rodrigues J, de la Torre Díez I, López-Coronado M, Saleem K (2015). Mobile-health: a review of current state in 2015. J Biomed Inform.

[21] Singh K, Drouin K, Newmark L, Rozenblum R, Lee J, Landman A, Pabo E, Klinger E, Bates D (2016). Developing a framework for evaluating the patient engagement, quality, and safety of mobile health applications. Issue Brief (Commonw Fund).

[22] Dou K, Yu P, Deng N, Liu F, Guan Y, Li Z, Ji Y, Du N, Lu X, Duan H (2017). Patients' acceptance of smartphone health technology for chronic disease management: a theoretical model and empirical test. JMIR Mhealth Uhealth.

[23] Yao Q, Liu F, Hou X (2018). Research on blood pressure health management system on mobile terminal. J Adv Oxid Technol.

[24] Thornton L, Kay-Lambkin F, Tebbutt B, Hanstock T, Baker A (2018). A mobile phone–based healthy lifestyle monitoring tool for people with mental health problems (MyHealthPA): development and pilot testing. JMIR Cardio.

[25] Worthen-Chaudhari L, McGonigal J, Logan K, Bockbrader MA, Yeates KO, Mysiw WJ (2017). Reducing concussion symptoms among teenage youth: Evaluation of a mobile health app. Brain Inj.

[26] Rivera-Romero O, Olmo A, Muñoz R, Stiefel P, Miranda M, Beltrán LM (2018). Mobile health solutions for hypertensive disorders in pregnancy: scoping literature review. JMIR Mhealth Uhealth.

[27] Arbour MW, Stec MA (2018). Mobile applications for women's health and midwifery care: a pocket reference for the 21st century. J Midwifery Womens Health.

[28] Villanueva J, Suarez M, Garmendia O, Lugo V, Ruiz C, Montserrat J (2017). The role of telemedicine and mobile health in the monitoring of sleep-breathing disorders: improving patient outcomes. Smart Homecare Technol Telehealth.

[29] Quercia K, Tran PL, Jinoro J, Herniainasolo JL, Viviano M, Vassilakos P, Benski C, Petignat P (2018). A mobile health data collection system for remote areas to monitor women participating in a cervical cancer screening campaign. Telemed J E Health.

[30] Xie X, Zhou W, Lin L, Fan S, Lin F, Wang L, Guo T, Ma C, Zhang J, He Y, Chen Y (2017). Internet hospitals in China: cross-sectional survey. J Med Internet Res.

[31] Ahmed T, Lucas H, Khan AS, Islam R, Bhuiya A, Iqbal M (2014). eHealth and mHealth initiatives in Bangladesh: a scoping study. BMC Health Serv Res.

[32] Wu H, Lu N (2017). Online written consultation, telephone consultation and offline appointment: an examination of the channel effect in online health communities. Int J Med Inform.

[33] Kamsu-Foguem B, Foguem C (2014). Telemedicine and mobile health with integrative medicine in developing countries. Health Policy Technol.

[34] Colaci D, Chaudhri S, Vasan A (2016). mHealth interventions in low-income countries to address maternal health: a systematic review. Ann Glob Health.

[35] Khatun F, Heywood AE, Ray PK, Bhuiya A, Liaw S (2016). Community readiness for adopting mHealth in rural Bangladesh: a qualitative exploration. Int J Med Inform.

[36] Hou J, Michaud C, Li Z, Dong Z, Sun B, Zhang J, Cao D, Wan X, Zeng C, Wei B, Tao L, Li X, Wang W, Lu Y, Xia X, Guo G, Zhang Z, Cao Y, Guan Y, Meng Q, Wang Q, Zhao Y, Liu H, Lin H, Ke Y, Chen L (2014). Transformation of the education of health professionals in China: progress and challenges. Lancet.

[37] Wu Q, Zhao L, Ye X (2016). Shortage of healthcare professionals in China. Br Med J.

[38] Hoque M (2016). An empirical study of mHealth adoption in a developing country: the moderating effect of gender concern. BMC Med Inform Decis Mak.

[39] Guo S, Guo X, Fang Y, Vogel D (2017). How doctors gain social and economic returns in online health-care communities: a professional capital perspective. J Manage Inf Syst.

[40] Mishra A, Anderson C, Angst C, Agarwal R (2012). Electronic health records assimilation and physician identity evolution: an identity theory perspective. Inf Syst Res.

[41] Dutta-Bergman M (2004). Complementarity in consumption of news types across traditional and new media. J Broadcast Electron Media.

[42] Dutta-Bergman M (2016). Interpersonal communication after 9/11 via telephone and internet: a theory of channel complementarity. New Media Soc.

[43] Rains S, Ruppel E (2013). Channel complementarity theory and the health information-seeking process: further investigating the implications of source characteristic complementarity. Commun Res.

[44] Lee E, Han S (2015). Determinants of adoption of mobile health services. Online Inf Rev.

[45] Mackert M, Mabry-Flynn A, Champlin S, Donovan E, Pounders K (2016). Health literacy and health information technology adoption: the potential for a new digital divide. J Med Internet Res.

[46] Zhang X, Han X, Dang Y, Meng F, Guo X, Lin J (2017). User acceptance of mobile health services from users' perspectives: the role of self-efficacy and response-efficacy in technology acceptance. Inform Health Soc Care.

[47] Guo X, Zhang X, Sun Y (2016). The privacy–personalization paradox in mHealth services acceptance of different age groups. Electron Commer Res Appl.

[48] Hossain MN, Okajima H, Kitaoka H, Ahmed A (2017). Consumer acceptance of eHealth among rural inhabitants in developing countries (a study on portable health clinic in Bangladesh). Procedia Comput Sci.

[49] Carroll JS, Payne JW (1976). The psychology of the parole decision process: a joint application of attribution theory and information-processing psychology. Cognition and Social Behavior.

[50] Sjovall AM, Talk AC (2004). From actions to impressions: cognitive attribution theory and the formation of corporate reputation. Corp Reputation Rev.

[51] Tongji Hospital.

[52] Guo X, Han X, Zhang X, Dang Y, Chen C (2015). Investigating m-health acceptance from a protection motivation theory perspective: gender and age differences. Telemed J E Health.

